# The impact of treatment delay on skin cancer in COVID-19 era: a case-control study

**DOI:** 10.1186/s12957-021-02468-z

**Published:** 2021-12-24

**Authors:** Konstantinos Seretis, Eleni Boptsi, Anastasia Boptsi, Efstathios G. Lykoudis

**Affiliations:** Department of Plastic Surgery, Medical School, University of Ioannina, Leoforos St. Niarchou, 45110 Ioannina, Greece

**Keywords:** Skin cancer, COVID-19, Melanoma, Squamous cell carcinoma

## Abstract

**Background:**

The outbreak of COVID-19 pandemic led to a 2-month lockdown in Europe. Elective surgeries, including skin cancer excisions, were postponed. The purpose of this prospective case-control study was to assess the impact of the treatment delay on patients with non-melanoma skin cancer (NMSC) or melanoma operated in the first post-lockdown period.

**Methods:**

A comparative study of skin cancer operations performed in a 4-month period either in 2020 or in 2019 was conducted. All data were collected from a prospectively maintained clinic database and the pathological reports. Continuous variables were compared with t test or Mann-Whitney *U* test according to their distribution. Categorical variables were compared with Fisher exact test. Odds ratio (OR) with 95% confidence interval (95% CI) was used to assess the risk of excising high-risk NMSC in 2020 compared with 2019.

**Results:**

Skin cancer excision was performed in 158 cases in 2020 compared to 125 cases in 2019 (26.4% increase). Significantly, more SCC were excised in 2020 (*p* = 0.024). No significant difference for several clinical parameters regarding BCC, SCC, and melanoma was identified. However, the reconstructive method applied, following NMSC excision, was significantly different, requiring frequently either skin grafting or a flap.

**Conclusion:**

These results indicate that skin cancer treatment delay, due to COVID-19 pandemic, is related to an increased incidence of SCC and more complicated methods of reconstruction. Considering the relapsing COVID-19 waves, significant skin cancer treatment delays should be avoided.

**Trial registration:**

The study adhered to the STROBE statement for case-control studies.

## Background

Coronavirus disease (COVID-19) has affected tremendously the daily lives and medical systems worldwide. On March 11, 2020, the coronavirus outbreak was declared a global pandemic by the World Health Organization. Then, most European countries imposed almost a complete lockdown, in efforts to limit the spread of the disease to their population and support effectively the national health systems. In that respect, elective surgery came to a halt, conserving vital medical resources, increasing ICU bed capacity, and protecting patients and healthcare workers from contracting the disease. This phase restricted also the access to the healthcare facilities, delayed treatments normally deemed as essential, and discouraged patients from seeking care [1].

More than 18 months since the first wave of the coronavirus and the associated lockdown, it is essential to realize its impact on the patients and their illnesses. This analysis is essential in order for the hospital structures and particularly the surgical sectors to be better organized, prioritize the surgical interventions, and prevent the most vulnerable population and those requiring surgery from suffering and even succumbing to their illnesses as “collateral damages” from the pandemic. Reviewing the literature, the data regarding the impact of the pandemic on the skin cancer management is scarce [2–4]. The aim of the current study was to evaluate the clinical features and surgical outcomes of patients with skin cancer operated in the post-lockdown period.

## Methods

A prospective comparative study was conducted in the Plastic Surgery Department of the Ioannina University Hospital, the only tertiary hospital of the prefecture of Epirus and one of the thirteen COVID-19 reference hospitals in Greece. The study protocol conformed to the ethical guidelines of the 1975 Declaration of Helsinki and adhered to the STROBE statement for case-control studies [5].

Patients with skin cancer, either non-melanoma (NMSC, namely, basal cell carcinoma-BCC or squamous cell carcinoma-SCC) or melanoma (MM), who were operated in a 4-month period, between the end of the lockdown and before the hospital restrictions of the second wave (20 May 2020 to 20 September 2020), were included in the study. Other types of cancer affecting the skin, precancerous and non-cancerous skin tumors, were excluded from the study. The 2020 cohort was compared with the cohort of skin cancer patients, operated in the same period in 2019. A prospectively maintained clinic database and the pathological reports were used to collect demographics, clinical, and surgical parameters of the study population.

Outcomes of interest were the number and types of skin cancers excised during the study periods, the skin cancer type characteristics, the reconstructive methods used, and the proportion of complete excision margins achieved.

Continuous variables were compared with t test or Mann-Whitney *U* test according to their distribution, whereas Fisher exact test was used for categorical variables. Odds ratio (OR) with 95% confidence interval (95% CI) was used to assess the risk of excising high-risk NMSC in 2020 compared with 2019. Statistical significance was defined as *p* **<** 0.05. Data were analyzed using SPSS (IBM SPSS Statistics for Macintosh, Version 26) [6].

## Results

**Fig. 1:**
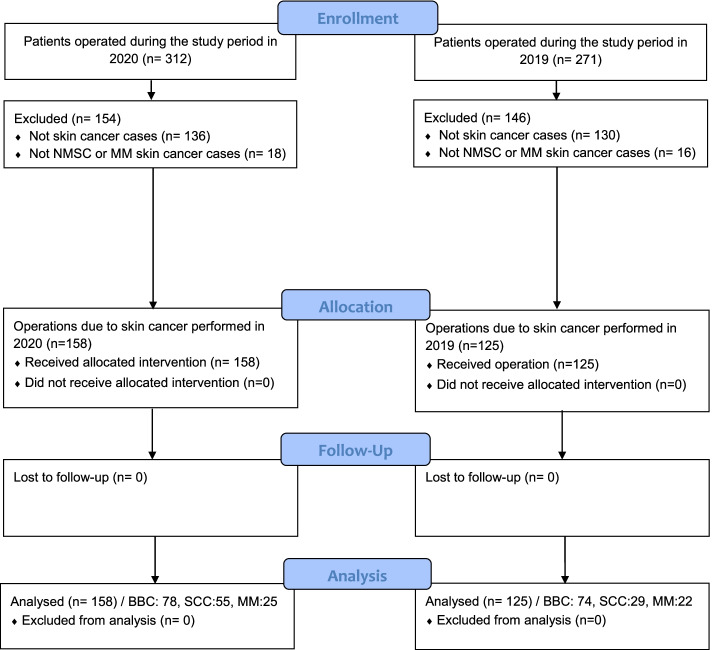
Study flow diagram

During the study period, 312 operations were performed for skin lesions in 2020 compared to 271 in 2019 (see Fig. 1, which demonstrates the study flow diagram). The inclusion criteria fulfilled 283 cases, 158 in 2020 and 125 in 2019 (26.4% increase). The patient demographic data are shown in Table 1. There were no significant differences between groups in terms of age and gender for NMSC. A greater number of females with MM were operated in 2020 (*p* < 0.05).

**Table 1:** Skin cancer patient characteristics

Variables	BCC		***p*** value	SCC		***p*** value	MM		***p*** value
	2020	2019		2020	2019		2020	2019	
**Patients (*n*)**	78	74	0.84	55	29	0.92	25	22	0.92
**Age (years)**	74.82 + 14.04	76.88 + 9.63		77.82 + 10.40	77.21 + 11.58		63.64 + 18.19	66.23 + 13.97	
**Age range (years)**	36‑99	53‑99		49‑94	52‑97		33‑90	43‑82	
**Gender**			0.38			0.98			**0.01**
Male (*n*)	43	46		40	21		11	18	
%	55.13	62.16		72.73	72.41		44	81.82	
Female (*n*)	35	28		15	8		14	4	
%	44.87	37.84		27.27	27.59		56	18.18	

The characteristics of the excised NMSC cases are reported in Table 2. Significantly, more SCCs were excised in 2020. There were no significant differences for NMSC, BCC, and SCC in several clinical parameters, such as NCCN risk group, the tumor size in terms of diameter measured by pathologist or the corresponding T category, SCC degree of differentiation, and negative margins achieved following excision. The OR of high-risk NMSC in 2020 compared with 2019 was 1.97 (95% CI, 0.38‑10.38). The type of reconstruction performed was significantly different for NMSC, requiring frequently either skin grafting or a flap (*p* = 0.006).

**Table 2:** Non-melanoma skin cancer characteristics

Variables	BCC		***p*** value	SCC		***p*** value	NMSC		***p*** value
	2020	2019		2020	2019		2020	2019	
**No. patients**	78	74		55	29		133	103	**0.04**
**NCCN risk group**			0.44			0.69			
Low	4	2		1	0				
High	74	72		27	13				
Very high				27	16				
**T category**			0.72			0.56			0.71
1	64	57		29	16		93	73	
2	12	14		19	9		31	23	
3	2	3		7	3		9	6	
4	0	0		0	1		0	1	
**Diameter (mm)**	12.45 + 8.67	13.48 + 9.48	0.50	22.00 + 18.56	23.75 + 18.13	0.47			
**Positive margin**			0.57			0.53			0.73
No	64	58		48	27		112	85	
Yes	14	16		7	2		21	18	
**Degree of differentiation**			-			0.73	-	-	-
1				21	9				
2				20	13				
3				14	7				
**Reconstruction type**^a^			**0.036**			0.14			**0.006**
Primary repair	22	33		11	10		33	43	
Skin grafting	17	15		16	8		33	23	
Flap	39	26		28	11		67	37	

*NCCN* National Comprehensive Cancer Network

^a^Primary repair compared to skin grafting/flap

The characteristics of the excised MM cases are reported in Table 3. No differences were found in the characteristics of excised MM or the type of operation performed. Significantly, thicker MMs were excised in 2019, even though the total number of primary excisions during the study period was low (5 in 2020 vs. 5 in 2019).

**Table 3:** Malignant melanoma characteristics

Variables	Melanoma		***p*** value
	2020	2019	
**Patients (*n*)**	25	22	
**Diameter (mm)**	9.71 + 10.53	17.13 + 14.61	0.07
**Breslow depth (*n*)**	8	5	**0.02**
**(mm)**	1.31 + 0.59	6.88 + 2.10	
**pT category**			**0.03**
0	1	3	
1	5	1	
2	2	0	
3	1	0	
4	0	4	
**Operation type**			0.95
Excisional biopsy	5	5	
Wide excision	10	7	
Wide excision + SLNB	7	7	
CLND	3	3	

*SLNB* sentinel lymph node biopsy, *CLND* complete lymph node dissection

## Discussion

The COVID-19 pandemic has radically altered the clinical and surgical daily routine, having a great impact on all aspects of health care and surgical practice, ranging from workforce, procedural prioritization [7] and risk of viral illness and transmission intraoperatively [8]. That had a huge impact on cancer care since proper screening and diagnosis were missed and therapies were interrupted. In Australia, a decline of 10% in cancer screening was reported, leading to approximately 2500 missed diagnoses during the lockdown [9]. A cross-sectional study, conducted at a maxillofacial surgery department, reported a decrease in the number of outpatients visits, hospitalizations, and tumor removals, which were halved [10]. Similarly, a decline even in the diagnosis of cardiovascular disease in the USA and therefore an increase in mortality rates were recorded [11]. These data, along with concerns that several high-risk patients for skin cancer postponed their scheduled appointment due to the fear of contagion, foreshadow a future cancer peak [9, 12, 13]. Therefore, with the COVID-19 pandemic appearing unlikely to resolve soon, there is a need to ensure and plan adequate and safe access to cancer care, while preventing considerable spread of the disease [14]. Several studies have showed minimal risk of COVID-19 infection or complication after elective surgery [15–18]. Teitelbaum et al. demonstrated that 5633 surgeries could be done without occurring serious cases of postoperative COVID-19. Following the safety protocols, plastic surgeons were able to work safely during the pandemic and thus postponing surgical procedures may be unnecessary [16].

Our findings delineate how the COVID-19 pandemic is associated with an increased skin cancer incidence and surgical workload after the lockdown termination. Although more skin cancer operations were performed in the post-lockdown period, this was significant only for SCC. In fact, postponing surgery by only 2 months led to almost a doubling in the number of SCC excised. Although a statistically significant worsening in terms of NMSC characteristics was not observed, the reconstructive modalities changed, requiring in the 2020 cohort mostly skin grafting or a flap. As regards to melanoma, the 2-month surgical delay did not seem to result in more advanced melanomas or type of operation performed.

In a retrospective case-control study, Valenti et al. compared 280 skin cancer excisions, performed from May to November 2020, to 265 in the same period in 2019 [19]. They reported a significantly higher number of advanced skin cancers after the end of the first lockdown in Italy, since they excised 54 advanced skin tumors, compared to 22 in 2019. They attributed the results to the delayed follow-up of patients with previously removed skin cancers. Canedo et al., in a retrospective cross-sectional study, reported that 18 new melanoma cases were diagnosed during April to August 2020 in comparison to 48 in the same period in 2019 [20]. More specifically, the decrease referred to in situ melanomas, while on the other hand, there was an increase in melanomas with over 2-mm thickness (38.9% in 2020 compared to 8.3% in 2019). In the same direction, Shannon et al. reported 298 melanoma cases after the termination of the lockdown, from June to August 2020, and 358 in the same period in 2019, with 153 and 172 being invasive melanomas, respectively [21]. Nonsignificant difference in thickness or pT staging was noted. However, 56 cases in 2020 and 68 in 2019 were surgically evaluated and an increase in tumor depth, proportion of tumors staged as pT3/pT4 (35.7% in 2020 and 19.1% in 2019) and satellitosis in patients after the lockdown was reported.

Tejera-Vaquerizo et al. constructed an exponential growth model for SCC and melanoma and estimated tumor size after 1, 2, and 3 months of potential surgical delay, suggesting that delaying SCC or melanoma treatment by 1 month or longer increases the proportion of more advanced cases [12]. More specifically, a 3 month-delay increases the proportion of T3 SCC by 72% and the melanoma with a Breslow thickness of > 6 mm by 30%. Ten-year disease-specific survival rates decreased significantly over the 3-month delay analyzed, to 65.6% for SCC and 65% for melanoma. Correlating this exponential model to our findings, concerning SCC, a considerable increase in more advanced tumors after the second and third wave of the pandemic is anticipated. This eventual SCC progress to a more advanced stage may be associated with higher mortality [22].

During the pandemic, the operations in the English health system (NHS), which can be deferred for 10‑12 weeks, are those without any negative consequences from this postponement. However, deciding which cases should be postponed, it is necessary to weigh the risk of a potential viral infection against the repercussion from a skin cancer progression [23]. Μelanoma and non-melanoma skin cancers are diagnosed mostly in patients older than 60 years, while immunosuppression is considered as a risk factor for skin cancer development [23]. The old age, immunosuppression, and other comorbidities are also risk factors associated with severe complications from COVID-19 [24]. Consequently, the stratification of patients is very important in order to prioritize the surgical services, offering optimal treatment, while minimizing the COVID-19 effects. In case of SARS-CoV-2 infection, data shows reduced 30-day postoperative mortality should surgery is delayed for at least 7 weeks from diagnosis, where possible [25].

The British Association of Plastic Surgery and NHS have provided an escalation policy for plastic surgical procedures [26]. Their recommendations, based on the COVID-19 prevalence, aim to minimize its effect and preserve the surgical workforce. With high prevalence, all elective surgeries should be stopped, and emergency surgery should be limited. In this regard, recurrent SCC and MM should be prioritized, and BCC lesions could be deferred [26]. The American College of Surgeons recommends also a patient prioritization based on the prevalence of the COVID-19 disease in the area and conservative therapies as non-surgical alternatives whenever possible [27].

A novel scale, the Plastic Surgery Acuity Scale, modified from the Elective Surgery Acuity Scale, aimed to simplify surgical decision-making regarding scheduling or postponing surgeries [28]. Most skin malignancy excisions could either be postponed or performed in the outpatient setting, unless the skin cancer lesion has a rapid progression or invades beyond the dermis.

Baumann et al., NCCN, and American College of Mohs Surgery recommend the delay of treatment for patients with T0-T1 melanoma up to 3 months, if no macroscopic residual disease is noted at biopsy [23, 29, 30]. In case of disease ≥T2, the excision can be delayed up to 3 months if biopsy margins are negative [23]. Our results are consistent with these guidelines and thus we confirm their applicability in the time frame of less than 3 months.

Baumann et al. reported that SCC treatment delay was associated with statistically significant tumor growth, but not with an increased risk of mortality [23]. As a result, for patients with SCC T1 or T2a stage, a treatment delay of 2 to 3 months is recommended, unless they are immunosuppressed, or the tumor grows rapidly. Patients with SCC ≥T2b should be prioritized, limiting the treatment delay to 1 to 2 months. If radiotherapy is required, it should either be delayed or modified to hypofractionated radiation in order to limit the number of sessions [23].

Concerning basal cell carcinoma, NCCN recommends delaying treatment at least for 3 months during a pandemic outbreak due to its minimal soft tissue invasiveness and rare metastatic capacity [23, 31]. This treatment delay may lead to an increase in the tumor size without oncological impact [23, 31]. Our results confirm the aforementioned recommendations, even if a higher workload and operative burden in the post-COVID era is anticipated.

Taking into consideration the outcomes of this study and the current guidelines mentioned, we assume not only an increase in the number of untreated skin cancers but also more complicated operation types and worse oncologic outcomes, because of the much longer duration of the lockdown during the second and the third wave of the pandemic compared to the first one. In this respect, policy makers should ensure proper and timely management of skin cancer patients. Currently, COVID-19 testing 24‑48 h before elective surgical procedures is applied to protect both patients and staff [8]. A regular oncological follow-up through telemedicine and virtual visits could be designed and scheduled for patients at highest risk of SARS-CoV-2 infection and those with higher risk of developing skin cancer. Thus, the risk of potential contagion could be eliminated, encouraging at the same time the regular screening for the early skin cancer detection [19].

Limitation of the study is the single-center data provided. However, this center pertains to the only tertiary hospital, covering an extended geographical region. The small sample size, regarding melanoma, prevents us from providing conclusive results. Despite these limitations, the rigorous methodology applied, providing homogenous groups with similar patient characteristics, limited the effect from potential confounding factors and increased the reliability of the study. In addition, the study design and the similar measures imposed in Europe enhanced the generalizability of the results, which confirm the current proposed guidelines and underline the importance of timely skin cancer diagnosis and treatment. Despite the current vaccination in progress, the pandemic has not been constrained yet; thus, further studies are anticipated, in order to provide definite guidelines.

## Conclusions

COVID-19 has deeply changed surgeon’s daily practice. Care of cancer patients in such treacherous times poses challenges regarding the optimal care offered, while preventing further spread of COVID-19. The study outcomes confirm the existing guidelines but raise concerns regarding the proper management of SCC and the reconstructive methods required for NMSC. Taking into consideration the outbreaks of the pandemic succeeding one another, the pressure on the health system will continue to increase. Therefore, hospitals should restructure to undertake the skin cancer cases, meanwhile ensuring adequate resources for the management of COVID-19 patients and patient’s safety.

## Data Availability

Not applicable.

## Abbreviations

COVID-19: Coronavirus disease 2019
NMSC: Non-melanoma skin cancer
SCC: Squamous cell carcinoma
BCC: Basal cell carcinoma
STROBE: Strengthening the Reporting of Observational Studies in Epidemiology
ICU: Intensive care unit
MM: Malignant melanoma
OR: Odds ratio
CI: Confidence interval
SPSS: Statistical Package for the Social Sciences
NCCN: National Comprehensive Cancer Network
NHS: National Health System
SARS-CoV-2: Severe acute respiratory syndrome coronavirus-2
